# Effectiveness of asfotase alfa for treatment of adults with hypophosphatasia: results from a global registry

**DOI:** 10.1186/s13023-024-03048-6

**Published:** 2024-03-08

**Authors:** Priya S. Kishnani, Gabriel Ángel Martos-Moreno, Agnès Linglart, Anna Petryk, Andrew Messali, Shona Fang, Cheryl Rockman-Greenberg, Keiichi Ozono, Wolfgang Högler, Lothar Seefried, Kathryn M. Dahir

**Affiliations:** 1 Department of Pediatrics, Duke University Medical Center, 2351 Erwin Road, Durham, NC 27710 USA; 2 Hospital Infantil Universitario Niño Jesús, IIS La Princesa, Universidad Autónoma de Madrid, CIBERobn, ISCIII, Madrid, Spain; 3 Paris-Saclay University, AP-HP and INSERM, Paris, France; 4 Alexion, AstraZeneca Rare Disease, Boston, MA USA; 5 University of Manitoba, Winnipeg, MB Canada; 6 Osaka University, Suita, Osaka Japan; 7 Johannes Kepler University Linz, Linz, Austria; 8 University of Würzburg, Würzburg, Germany; 9 Vanderbilt University Medical Center, Nashville, TN USA

## Abstract

**Background:**

Hypophosphatasia (HPP) is a rare inherited disease caused by deficient activity of tissue-nonspecific alkaline phosphatase. Many adults with HPP have a high burden of disease, experiencing chronic pain, fatigue, limited mobility, and dental issues, contributing to decreased health-related quality of life (HRQoL). HPP may be treated with the enzyme replacement therapy asfotase alfa though real-world data in adults are limited. This analysis was conducted to assess the clinical effectiveness of asfotase alfa among adults in the Global HPP Registry.

**Methods:**

The Global HPP Registry is an observational, prospective, multinational study. Adults ≥ 18 years of age were included in this analysis if they had serum alkaline phosphatase (ALP) activity below the age- and sex-adjusted reference ranges, and/or *ALPL* variant(s), and received asfotase alfa for ≥ 6 months. Mobility was assessed with the 6-Minute Walk Test (6MWT), and patient-reported outcomes tools were used to assess pain (Brief Pain Inventory-Short Form), quality of life (36-item Short Form Health Survey, version 2 [SF-36v2]), and disability (Health Assessment Questionnaire-Disability Index) at multiple time points from baseline through Month 36. Data were collected as per usual standard of care; patients may not have contributed data at all time points.

**Results:**

A total of 190 patients met the inclusion criteria. For patients with ≥ 1 follow-up measurement, the mean distance achieved on 6MWT increased from 404 m (range 60–632 m) at baseline (n = 31) to 484 m at Month 12 (range 240–739 m; n = 18) and remained above baseline through Month 36 (n = 7). Improvements in mean self-reported pain severity scores ranged from − 0.72 (95% CI: − 1.23, − 0.21; n = 38) to − 1.13 (95% CI: − 1.76, − 0.51; n = 26) and were observed at all time points. Improvements in the Physical Component Summary score of SF-36v2 were achieved by Month 6 and sustained throughout follow-up. There was a trend toward improvement in the Mental Component Summary score of SF-36v2 at most time points, with considerable fluctuations from Months 12 (n = 28) through 36 (n = 21). The most frequent adverse events were injection site reactions.

**Conclusions:**

Adults with HPP who received asfotase alfa for ≥ 6 months experienced improvements in mobility, physical function, and HRQoL, which were maintained over 3 years of follow-up.

Registration: NCT02306720; EUPAS13514.

**Supplementary Information:**

The online version contains supplementary material available at 10.1186/s13023-024-03048-6.

## Introduction

Hypophosphatasia (HPP) is a rare inherited disease caused by deficient activity of tissue-nonspecific alkaline phosphatase (TNSALP) [1, 2]. Deficiencies in TNSALP result in accumulation of inorganic pyrophosphate, pyridoxal 5ʹ-phosphate (the circulating form of vitamin B_6_), and phosphoethanolamine [1–4]. Accumulation of extracellular pyrophosphate results in poor mineralization of bones and teeth, with related sequelae including fractures or pseudofractures, bone deformities, and dental complications, including premature loss of teeth [1, 2, 5, 6]. Hypercalcemia, hyperphosphatemia, and hypercalciuria can be present and associated with nephrocalcinosis, which may result in renal insufficiency, and ectopic calcification of ligaments, eyes, and other tissues. The burden of disease associated with HPP in adults is variable, with patients often experiencing combinations of symptoms, including chronic pain, fatigue, muscle weakness, limited mobility, fractures, pseudofractures, and dental abnormalities and reporting decreased health-related quality of life (HRQoL) [5, 7, 8]. Because of the heterogeneous nature of HPP, nonspecific nature of symptoms, and variation in disease severity, with manifestation at different ages, correct diagnosis can often be delayed [2, 9].

Asfotase alfa (Strensiq^®^; Alexion, AstraZeneca Rare Disease, Boston, MA, USA) is a human recombinant TNSALP enzyme replacement therapy (ERT) approved for the treatment of perinatal/infantile- and juvenile-onset HPP; in Japan, asfotase alfa is approved for all patients with HPP, regardless of age at onset. Clinical studies with 27 patients receiving asfotase alfa, predominantly adults with pediatric-onset HPP, have demonstrated improved physical function and HRQoL during up to 5 years of asfotase alfa treatment [10, 11]. Asfotase alfa was well tolerated in these studies; injection-site reaction was the most common treatment-emergent adverse event.

The Global HPP Registry has helped further our understanding of HPP given the disease’s rarity and heterogeneity [5, 8, 9, 12, 13]. The objective of this analysis was to assess the effectiveness of asfotase alfa on measures of mobility, functional status, pain, and HRQoL in a cohort of adults in the Global HPP Registry who received the drug.

### Methods

#### Data collection

The ongoing Global HPP Registry is an observational, prospective, multinational study initiated in 2014 (NCT02306720; EUPAS13514). The registry is sponsored by Alexion, AstraZeneca Rare Disease (Boston, MA, USA) and overseen by a scientific advisory board of medical experts, including employees of Alexion [9]. The protocol for the registry was approved by the institutional review board (or equivalent) at each participating site and is being conducted in accordance with European Medicines Agency Good Pharmacovigilance Practices, International Society for Pharmacoepidemiology Guidelines for Good Pharmacoepidemiology Practices, and the Declaration of Helsinki [9]. All patients or parent/legal guardians provided written informed consent and approval to release medical records for inclusion in the registry.

At the time of enrollment in the registry, and at the time of initiation of asfotase alfa (if different), patients’ HPP history, including clinical course, signs, symptoms, and/or complications, as well as burden of disease, were collected under conditions of routine clinical care in a real-world setting [9]. HPP manifestations were assigned to the appropriate body system/category (ie, skeletal, muscular, dental, neurologic, renal, constitutional/metabolic, rheumatologic, respiratory, or pain).

#### Study population

Patients of all ages diagnosed with HPP with diagnostic data available (serum alkaline phosphatase [ALP] activity below the age- and sex-adjusted reference ranges and/or an *ALPL* variant) were eligible for participation in the registry [9]. Patients were included in the study population for this analysis if they had initiated treatment with asfotase alfa at ≥ 18 years of age, had ≥ 6 months of exposure, and had provided date of informed consent, date of birth (or age at enrollment in countries that did not permit collection of birth date), and sex [9]. The requirements for the safety population were the same as those for the overall study population, except there was no requirement for minimum exposure to asfotase alfa.

#### Assessments

Mobility was assessed with the 6-Minute Walk Test (6MWT) [14], which has been validated as a reliable indicator of physical function in patients with HPP [15]. The use of mobility aids was allowed during the test, and their use was recorded. For patients who did not perform the 6MWT, such as those who were nonambulatory, the reason was not collected. Ambulatory status was not collected. HRQoL was assessed using the 36-item Short Form Health Survey, version 2 (SF-36v2) [16, 17]. Disability was assessed using the Health Assessment Questionnaire–Disability Index (HAQ-DI) [18]. Pain was self-reported using the Brief Pain Inventory-Short Form (BPI-SF) survey [19]. Additional details describing these assessments can be found in the Supplementary Appendix (See Additional file 1 for all supplementary material).

Targeted events of interest (including injection site reactions, injection-associated reactions, and severe hypersensitivity reactions) and serious adverse events that occurred on or after the first dose of asfotase alfa, or events occurring after enrollment for those who were already taking the drug, were reported. Data on targeted events of interest were not available for patients enrolled in Japan as data were collected in a separate database; however, serious adverse event data were merged with the global registry data.

#### Statistical analysis

Data were summarized using descriptive statistics. The data available for analysis for the different measures varied since data in the registry were obtained under conditions of routine clinical care in real-world settings. The COVID-19 pandemic may also have had an impact on data collection.

Data on available endpoints were analyzed both cross-sectionally and longitudinally at each time point of interest. In cross-sectional analyses, all patients for whom data were available at a specific time point are included. No statistical significance testing was performed on the cross-sectional data. For analyses of change from baseline, only patients with a pretreatment baseline value and at least 1 follow-up were included; for each time point, only patients with data at that time point were included in the change from baseline analysis. Change from baseline values are presented as mean (95% confidence interval [CI]) for all patients with data at that time point; for simplicity, baseline values presented are for all patients included in the change from baseline analysis.

Continuous variables are reported as median (minimum, maximum) and mean (standard deviation), as appropriate. Statistical significance testing was performed for comparisons from baseline to Months 6, 12, 24, and 36 using paired *t*-tests and Wilcoxon rank sum tests, as appropriate.

## Results

### Study population

**Fig. 1:**
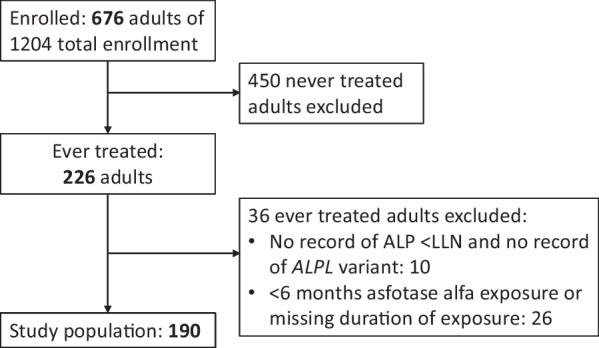
Study population. ALP, alkaline phosphatase; LLN, lower limit of normal

As of December 12, 2022, the Global HPP Registry contained 1204 patients, including 676 adults. Of these, 226 adults had ever been treated with asfotase alfa, and 190 of these adults met the inclusion criteria for this analysis (Fig. 1). The main reasons for exclusion were < 6 months exposure to asfotase alfa among those with documented low ALP and/or *ALPL* variant (n = 26) or with no record of either low ALP or an *ALPL* variant (n = 10). Demographics and baseline characteristics of all 190 included patients are summarized in Table 1. Median age at the start of treatment was 45.5 (min, max: 18.3, 77.9) years, 68.4% of patients were female, and 91.1% had pediatric-onset HPP. Seventeen patients were classified as having adult-onset HPP. The majority of patients had heterozygous or compound heterozygous gene mutations; 4 patients (2.1%) were homozygous. Patients had significant burden of disease with a median of 5 (range: 1–14) separate signs and symptoms involving a median of 3 (range: 1–8) body systems. The median duration of treatment with asfotase alfa was 3.1 years (range: 0.5–11.3). Supplementary Table 1 summarizes characteristics of the 9 patients who had low ALP but no confirmatory *ALPL* variant. In 6 patients, genetic testing was not performed, in some cases because the patient declined testing; 2 patients were negative for an *ALPL* variant, and 1 patient had the testing performed but the result was not available. All of these patients had low ALP levels and a constellation of signs and symptoms very suggestive of HPP. Eight of the 9 patients had fractures. Both patients with negative genetic test results had metatarsal and/or femoral fractures.

**Table 1:** Demographics and Baseline Characteristics of Study Patients

Characteristic	Study Population (N = 190)
Sex, n (%)	
Female	130 (68.4)
Male	60 (31.6)
Age at diagnosis of HPP, years	
N	159
Median (min, max)	37.1 (0, 77)
≤ 18 years at diagnosis, n (%)	32 (16.8)
> 18 years at diagnosis, n (%)	127 (66.8)
Missing, n (%)	31 (16.3)
Age at start of asfotase alfa treatment, years	
N	190
Median (min, max)	45.5 (18.3, 77.9)
Time from diagnosis to start of asfotase alfa treatment, years	
N	154
Median (min, max)	1.4 (0.01, 60.8)
White race, n (%)	153 (80.5)
Not Hispanic or Latino, n (%)	146 (76.8)
Number of signs and symptoms per patient	
N	180
Median (min, max)	5 (1, 14)
Number of body systems impacted per patient	
N	180
Median (min, max)	3 (1, 8)
History of signs and symptoms, n (%)	
Skeletal	120 (63.2)
Neurologic	30 (15.8)
Constitutional/metabolic	88 (46.3)
Muscular	71 (37.4)
Dental	129 (67.9)
Respiratory	7 (3.7)
Renal	26 (13.7)
Rheumatic	21 (11.1)
Pain	138 (72.6)
HPP onset, n (%)	
Pediatric	173 (91.1)
Perinatal/infantile onset	18 (9.5)
Juvenile onset	117 (61.6)
Pediatric onset, specific type unknown	38 (20.0)
Adult-onset HPP	17 (8.9)
Gene classification, n (%)	
Heterozygous	113 (59.5)
Compound heterozygous	55 (28.9)
Homozygous	4 (2.1)
Unknown/missing	18 (9.5)

*HPP* hypophosphatasia

The majority of patients with data for this analysis started treatment with asfotase alfa at a cumulative dose of 6 mg/kg per week (n = 137/179; 76.5%), either as 1 mg/kg 6 times per week or 2 mg/kg 3 times per week. A small number of patients started treatment at a dose of < 6 mg/kg per week (n = 35/179; 19.6%) or > 6 mg/kg per week (n = 7/179; 3.9%). There was little change in these dose levels overall at latest follow-up.

### Mobility

**Fig. 2:**
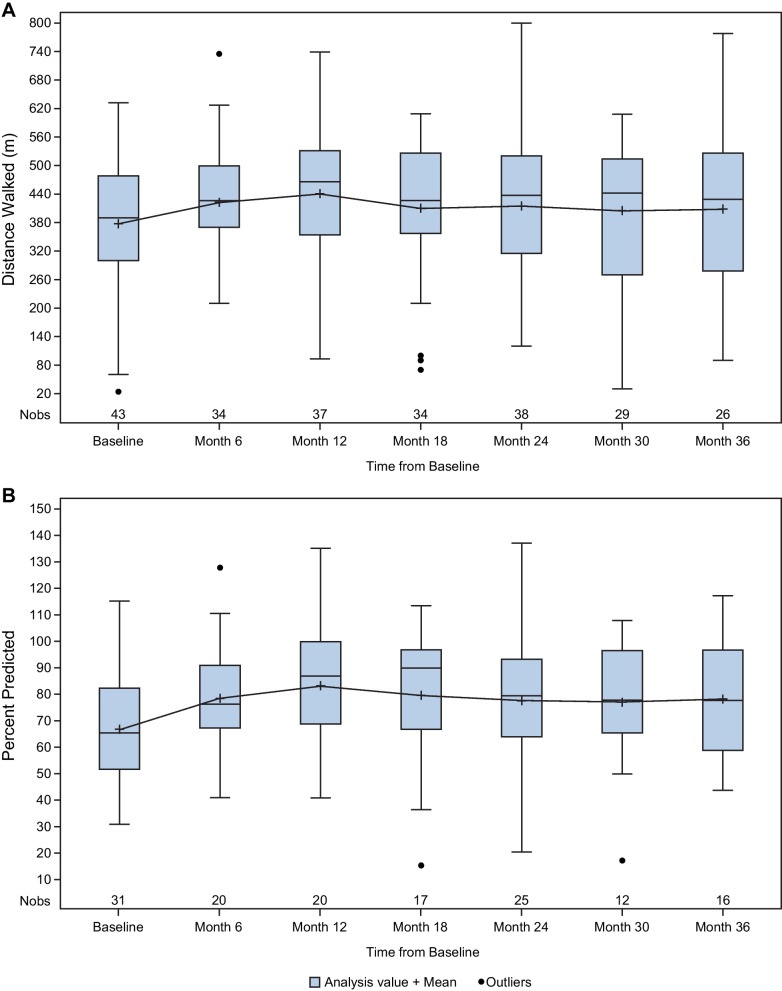
Cross-sectional analysis of 6MWT over time. (**A**) Total distance walked; (**B**) Percent predicted. 6MWT, 6-Minute Walk Test; Nobs, number of observations

At baseline, for patients with at least 1 follow-up measurement (n = 31), the mean 6MWT distance walked was 404 m, with 5 of 31 patients using an assistive device. The distribution of change in distance walked and percent predicted distance covered over the course of treatment with asfotase alfa in the cross-sectional analysis are shown in Fig. 2. In the pairwise analysis, 6MWT distance walked improved over 36 months of follow-up and was statistically significant at Month 12 (+ 93 m [95% CI: 47, 138; *P* < 0.001]; n = 18) and Month 24 (+ 62 m [95% CI: 0.4, 124; *P* = 0.049]; n = 15) (Supplementary Fig. 1A). At Month 36, the improvement in 6MWT was a mean + 45 m (95% CI: − 83, 172 [*P* = 0.42]; n = 7). The percent predicted distance achieved, normalized for sex and age relative to a healthy individual, was 73.9% at baseline. In the pairwise analysis, percent predicted distance ranged from 76.1% to 90.4% during follow-up and was most pronounced at Month 12 (change: 12% [95% CI: 2, 22; *P* = 0.02]; n = 10; Supplementary Fig. 1B).

### Pain

**Fig. 3:**
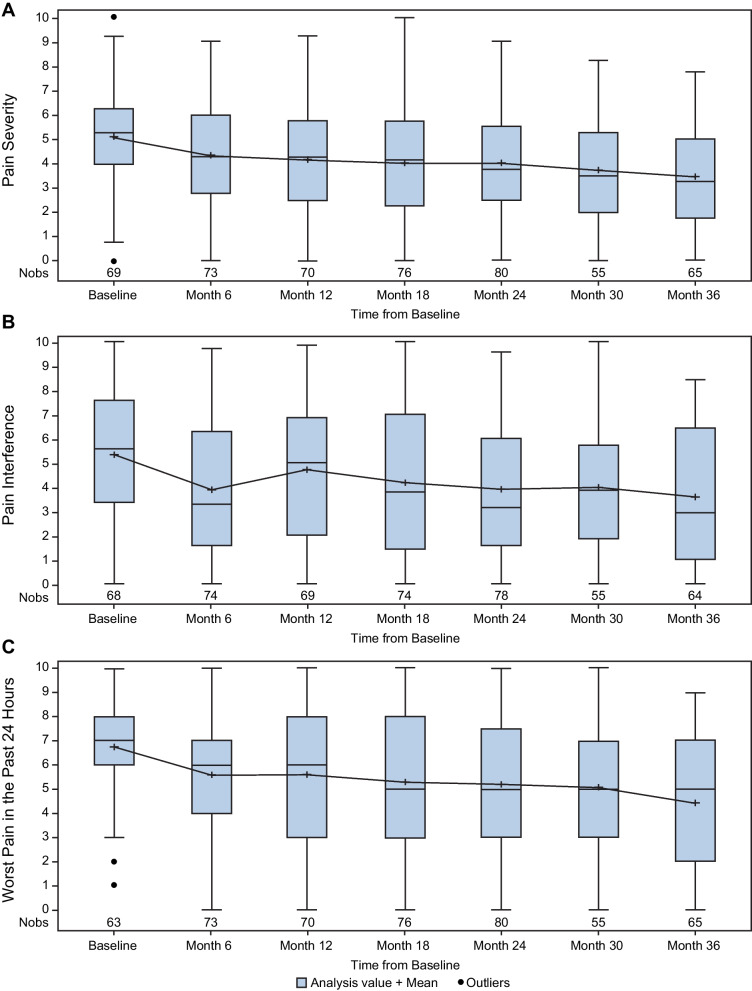
Cross-sectional analysis of self-reported pain measured with BPI-SF over time. (**A**) Pain severity; (**B**) Pain interference; and (**C**) Worst pain in past 24 h. BPI-SF, Brief Pain Inventory–Short Form; Nobs, number of observations

Distributions of changes in the components of BPI-SF (pain severity, pain interference, and worst pain in the past 24 h) are shown in Fig. 3 (cross-sectional analysis) and Supplementary Fig. 2 (pairwise comparison). Statistically significant improvements in mean self-reported pain severity scores, ranging from − 0.72 (95% CI: − 1.23, − 0.21; n = 38) to − 1.13 (95% CI: − 1.76, − 0.51; n = 26), were observed at all time points in the pairwise comparison (*P* ≤ 0.012). Pain interference scores improved by a range of − 0.87 (95% CI: − 1.85, 0.11; n = 23) to − 1.47 (95% CI :− 2.32, − 0.62; n = 26) during follow-up (*P* ≤ 0.005 through 24 months in the pairwise comparison). There was a statistically significant improvement at all time points tested in the pairwise comparison for the worst pain in the past 24 h (*P* ≤ 0.006), with improvements ranging from − 0.92 (95% CI: − 1.56, − 0.28; n = 38) to − 1.69 (95% CI: − 2.58, − 0.81; n = 26).

### Quality of life

**Fig. 4:**
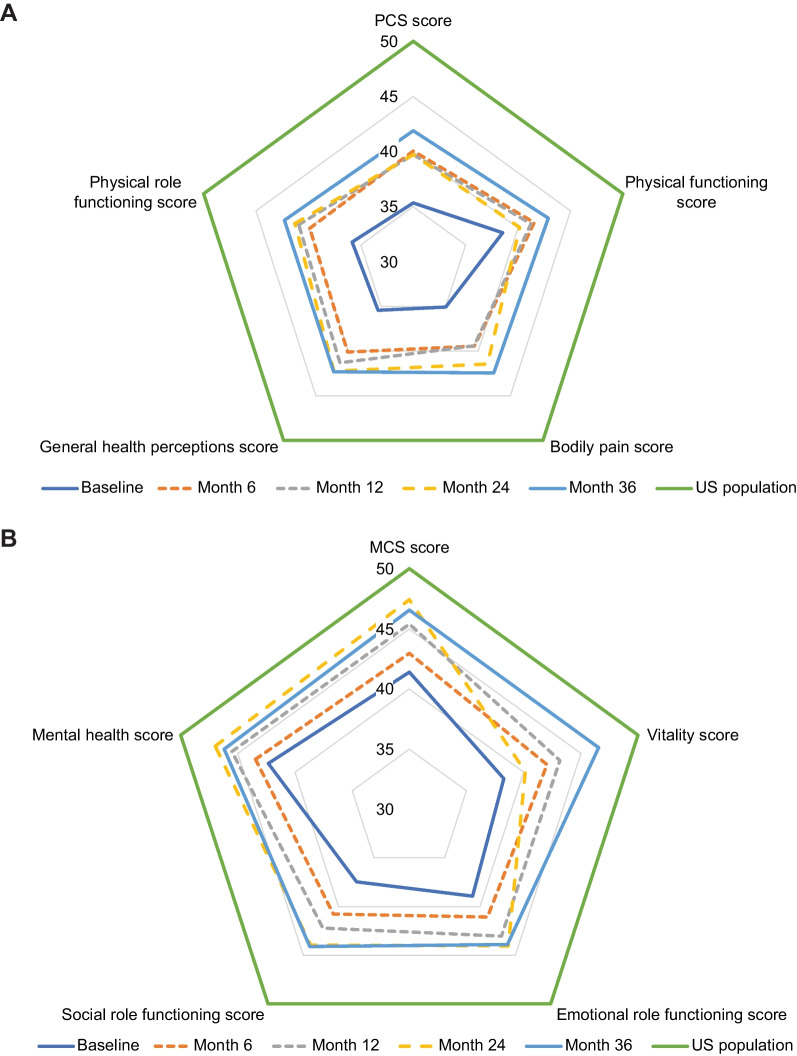
Cross-sectional analysis of quality of life measured with SF-36v2 over 36 months. (**A**) RADAR plot showing mean PCS score and its component domains; (**B**) RADAR plot showing mean MCS score and its component domains. MCS, Mental Component Summary; PCS, Physical Component Summary; SF-36v2, 36-item Short Form Health Survey, version 2

Figure 4 illustrates the change in all domains of SF-36v2 during 36 months of follow-up in the cross-sectional analysis. In the pairwise comparison, mean improvements from a mean baseline score of 35.7 (n = 48) were observed for the Physical Component Summary (PCS) score at Month 6 (change: 4.68 [95% CI: 0.16, 9.21; *P* = 0.043]; n = 34) and were numerically higher through Month 36, although not statistically significant beyond Month 12 (Supplementary Fig. 3A). Median changes were similar to mean changes.

Similarly, mean changes in physical functioning score (change: 4.10 [95% CI: 0.20, 8.00; *P* = 0.04]), physical role functioning score (change: 3.69 [95% CI: 0.19, 7.18; *P* = 0.039]), and bodily pain score (change: 4.52 [95% CI: 0.34, 8.70; *P* = 0.035]; n = 28) were statistically significant after 12 months of treatment (n = 28), but changes did not reach statistical significance through 36 months. Improvements in mean general health perceptions were observed at Month 6 (change: 3.96 [95% CI: 0.17, 7.75; *P* = 0.041]; n = 34) and sustained through Month 36.

There was a trend toward improvement in the Mental Component Summary (MCS) score at most time points, with considerable fluctuations between Months 12 and 36 (Supplementary Fig. 3B). Statistically significant improvements were noted in mean social role functioning, improving from baseline by 4.48 at Month 12 (95% CI: 0.72, 8.23 [*P* = 0.021]; n = 28), and in the mental health score, increasing from baseline by 4.64 points at Month 24 (95% CI: 0.59, 8.69 [*P* = 0.027]; n = 22).

### Disability

**Fig. 5:**
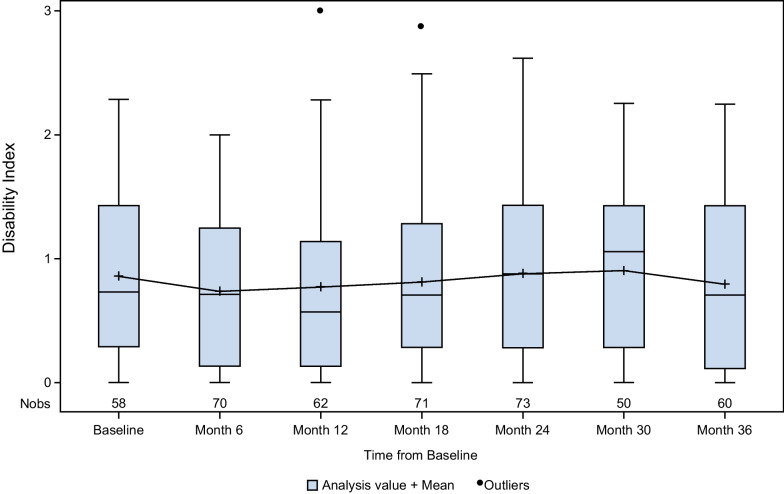
Cross-sectional analysis of disability measured with HAQ-DI over time. HAQ-DI, Health Assessment Questionnaire–Disability Index; Nobs, number of observations

Figure 5 shows disability scores (HAQ-DI) during 36 months of follow-up for all patients with data at any time point. Disability scores were unchanged over time. The proportion of patients with a HAQ-DI score of 0, indicating no disability, increased from 8.6% (n = 5) at baseline to 18.3% (n = 11) at Month 36.

### Safety

A total of 216 patients were included in the safety analysis. Targeted events of interest (both serious and nonserious) regarded by the investigator as related to treatment with asfotase alfa that occurred on treatment or within 30 days of drug discontinuation and occurring in more than 1 patient were injection site reactions (n = 26; 12.0%), injection-associated reactions (n = 12; 5.6%), and severe hypersensitivity reaction (n = 2; 0.9%). A total of 11 serious adverse events regarded by the investigator as related to asfotase alfa occurred in 7 patients, with none occurring in more than 1 patient.

## Discussion

Previous studies of the clinical effectiveness of asfotase alfa have been limited to small numbers of patients from single countries [10, 11]. Here we describe the real-world effectiveness of asfotase alfa through both functional measures and patient-reported outcomes (PROs) in a cohort of 190 adult patients enrolled in the Global HPP Registry (the largest studied to date) who met inclusion criteria. Patient mobility was increased following initiation of asfotase alfa during 36 months of follow-up; the distance achieved on the 6MWT increased by up to 93 m, representing an almost 25% increase in mobility. This improvement in mobility is consistent with results from a single-center study of 14 adults with pediatric-onset HPP in which a significant increase in 6MWT of 53 m after 12 months of treatment with asfotase alfa (*P* = 0.023) was observed [11] and helps to further build upon our knowledge of this rare disease. Although these improvements were not statistically significant at all time points, undoubtedly influenced by the number of patients with available measurements at each time point, the distances achieved were substantially greater than the estimated minimum clinically important difference (MCID) of 31.1 m for 6MWT in adults with HPP [15].

Consistent improvements in HRQoL and pain were observed in this study. Improvements in PCS of SF-36v2 ranged from 4.61 to 8.12 during 36 months of treatment with asfotase alfa, and improvements in MCS ranged from 1.72 to 5.28 at 12 to 36 months after starting treatment. These changes are consistent with those in an earlier single center study, which reported increases in median PCS from 26 to 33 and median MCS from 53 to 56 over 12 months [11]. Although the MCID for the PCS or MCS score has not been established in HPP, improvements in PCS scores were consistently greater than the MCID established for other conditions (3.9 in degenerative cervical myelopathy, 4.1 in distal radius fracture, and 3.3 in patients with osteoarthritis of the lower extremities) [20–22]. Consistent improvements in pain severity, pain interference with activities of daily living, and worst pain in the past 24 h, as measured using BPI-SF, were seen over the course of follow-up. Similar results were reported during 5-year follow-up to a phase 2 randomized, open-label, trial of asfotase alfa [10]. There was no change in disability as measured using the HAQ-DI, although the increase in the number of patients with no disability is encouraging. However, changes in HAQ-DI have not previously been studied in patients with HPP, so its utility in this population is still being determined. One possibility for the lack of effect of treatment on HAQ-DI is that HPP has little effect on the activities of daily living assessed with this instrument in most patients. Overall, however, the consistent improvements observed across a range of instruments indicate an important improvement in overall health status in patients treated with asfotase alfa.

This study has a number of limitations. This was an observational registry of patients undergoing standard-of-care treatment, so data were not routinely collected for all patients at prescribed time points. While patients with pediatric- and adult-onset HPP were evaluated, including 9.5% with perinatal/infantile HPP, greater than 90% of patients had pediatric-onset HPP, which may affect the generalizability of the results. In addition, a small percentage of patients had no genetic confirmation of HPP (9/190, 4.7%) because genetic testing was not performed, or *ALPL* variants were not identified, or because the result was missing. However, all of these patients had low ALP levels and a constellation of signs and symptoms very suggestive of HPP. The inclusion of these patients without genetically confirmed HPP reflects the real-world nature of a global registry, and their indication for ERT was determined at the point of care of their respective treating physicians, again reflecting practices in the clinical setting. Because data were taken from an international registry, regional variations in care may influence the timing, number, and types of assessments routinely performed in HPP in different countries, as well as indication for and timing of initiation of asfotase alfa treatment. As a result, effectiveness assessments were available for only a minority of patients and patients contributed data at different time points. While the registry also includes data from untreated patients with HPP, the differences in baseline disease burden did not allow a direct comparison between the groups. Instead, the clinical benefit was assessed through pairwise comparisons between pretreatment baseline values and follow-up assessments on treatment. Another limitation is that the ambulatory status of these patients is not known. As a result, it is not clear whether missing data for the 6MWT were due to the test not being performed or because the patient was unable to perform it; this likely affected interpretation of both mobility and disability data. Finally, while each of the PROs in this analysis are regarded as clinically relevant, the MCIDs for these measures in HPP are unknown except for the MCIDs for the 6MWT.

## Conclusions

Asfotase alfa was associated with improvements in mobility, HRQoL measures, and pain, as early as 6 months and most notably at 12 months of treatment. Improvements in mobility, pain, and HRQoL appeared to be clinically relevant. Asfotase alfa was generally well tolerated, with the most common adverse events related to treatment being injection site reactions. ERT with asfotase alfa demonstrated sustained effectiveness in patients with HPP and was associated with overall improvements in health status.

### Supplementary Information

**Additional file 1**. Additional results of assessments and individual patient characteristics recorded in the Global HPP Registry.

## Data Availability

Alexion, AstraZeneca Rare Disease will consider requests for disclosure of clinical study participant-level data provided that participant privacy is assured through methods like data de-identification, pseudonymization, or anonymization (as required by applicable law), and if such disclosure was included in the relevant study informed consent form or similar documentation. Qualified academic investigators may request participant-level clinical data and supporting documents (statistical analysis plan and protocol) pertaining to Alexion-sponsored studies. Further details regarding data availability and instructions for requesting information are available in the Alexion Clinical Trials Disclosure and Transparency Policy at https://alexion.com/our-research/research-and-development. Link to Data Request Form: https://alexion.com/contact-alexion/medical-information.
