# Supplementary material for: Effectiveness of asfotase alfa for treatment of adults with hypophosphatasia: results from a global registry
Source: Orphanet J Rare Dis. 2024 Mar 8;19:109. doi: 10.1186/s13023-024-03048-6 (PMC10921796; doi:10.1186/s13023-024-03048-6)
Supplement: Supplementary file 1 — Additional file 1. Additional results of assessments and individual patient characteristics recorded in the Global HPP Registry. [file 13023_2024_3048_MOESM1_ESM.docx]

**Supplementary Appendix**

**Effectiveness of Asfotase Alfa for Treatment of Adults With Hypophosphatasia: Results From a Global Registry**

Priya S. Kishnani, Gabriel Ángel Martos-Moreno, Agnès Linglart, Anna Petryk, Andrew Messali, Shona Fang, Cheryl Rockman-Greenberg, Keiichi Ozono, Wolfgang Högler, Lothar Seefried, Kathryn M. Dahir

Corresponding author: Priya S. Kishnani (Priya.Kishnani@duke.edu)

**Appendix**

### Mobility was assessed through the 6-Minute Walk Test (6MWT), which measures the impact of pulmonary, cardiovascular, and musculoskeletal function and fatiguability on ambulatory capacity by measuring the distance a person can walk on a hard, flat surface over a period of 6 minutes [14]. The 6MWT has been validated as a reliable indicator of physical function in patients with HPP [15]. The use of mobility aids was allowed during the test and was recorded. For patients who did not perform the 6MWT, such as those who were nonambulatory, the reason was not collected.

### Health-related quality of life (HRQoL) was assessed using the 36-item Short Form Health Survey, version 2 (SF-36v2) [16,17]. The SF-36v2 (QualityMetric Inc, Lincoln, RI, USA) is a generic 36-item questionnaire that measures HRQoL via scores in 8 domains: vitality, physical functioning, bodily pain, general health perceptions, physical role functioning, social role functioning, emotional role functioning, and mental health. Each domain has 2 to 10 items, with a maximum total score of 100; lower scores indicate worse health status. Scores for the individual sub-domains, including the Physical Component Summary (PCS) score and Mental Component Summary (MCS) score are normalized to be reported out of 100.

### Disability was assessed using the Health Assessment Questionnaire–Disability Index (HAQ-DI) [18]. The HAQ-DI consists of 20 items and measures physical ability in 8 categories: dressing, arising, eating, walking, hygiene, reach, grip, and usual activities. An index score indicating overall disability is provided, with scores ranging from 0 to 3, with 0 representing function without any difficulty and 3 representing inability to do the task.

### Pain was self-reported using the Brief Pain Inventory-Short Form (BPI-SF) survey [19]. Pain severity, pain interference with daily activities, and worst pain in the last 24 hours were all scored on a scale from 0 to 10, with lower scores representing less pain.

**Supplementary Figure 1.** Change from baseline in 6MWT distance over time for patients with baseline and at least 1 follow-up assessment. (**A**) Mean (95% CI) total distance walked; (**B**) Mean (95% CI) percent predicted.

**A**

**B**

6MWT, 6-Minute Walk Test; CI, confidence interval.

**Supplementary Figure 2.** Mean change from baseline in BPI-SF domains over 36 months (population with baseline assessment and at least 1 follow-up assessment).


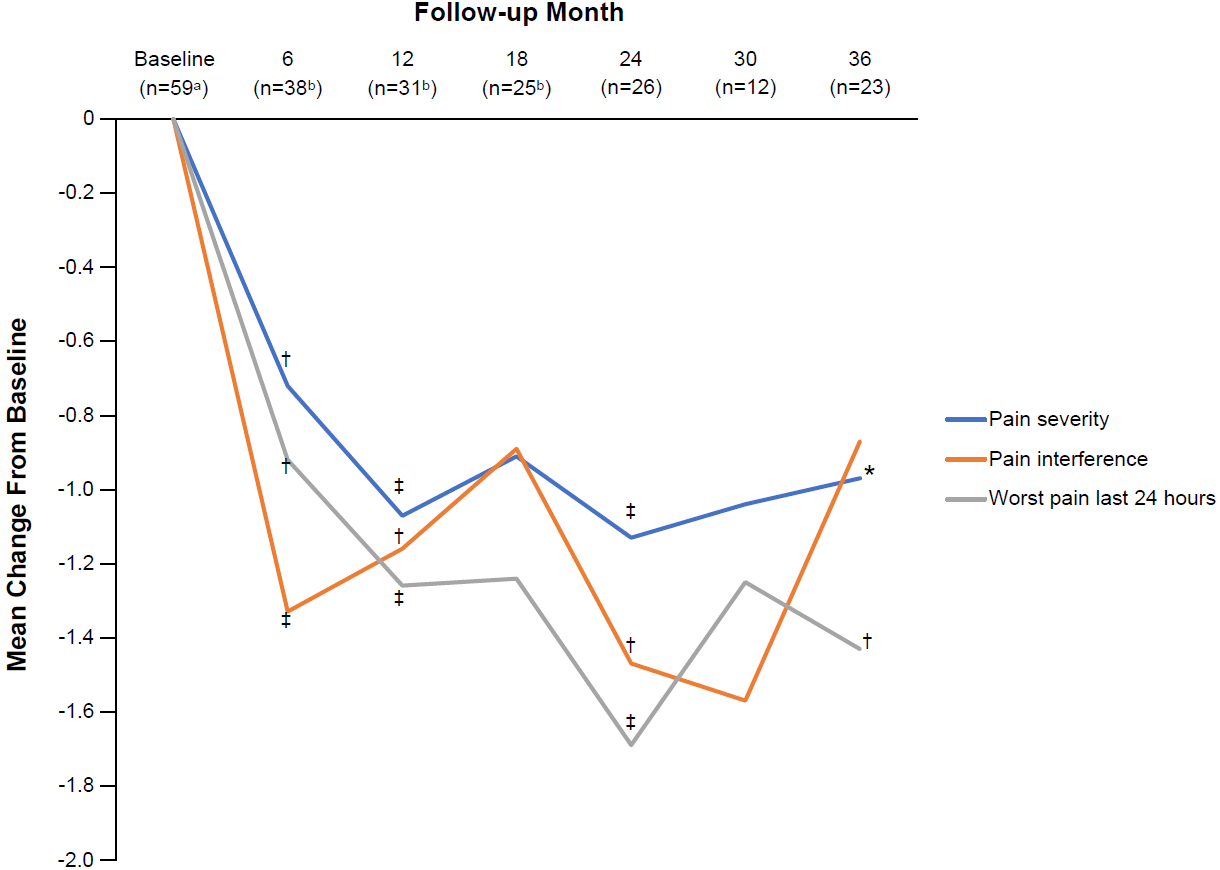


^a^Only 53 patients provided valid baseline values for worst pain in last 24 hours.

^b^The number of patients with valid data for pain severity at 6, 12, and 18 months was 39, 30, and 24, respectively.

**P*<0.05; ^†^*P*<0.01; ^‡^*P*≤0.001.

BPI-SF, Brief Pain Inventory–Short Form.

**Supplementary Figure 3.** Mean change from baseline in quality-of-life scores over 36 months for SF-36v2 domains (population with baseline assessment and at least 1 follow-up assessment). (**A**) PCS score and its component domain scores; (**B**) MCS score and its component domain scores.


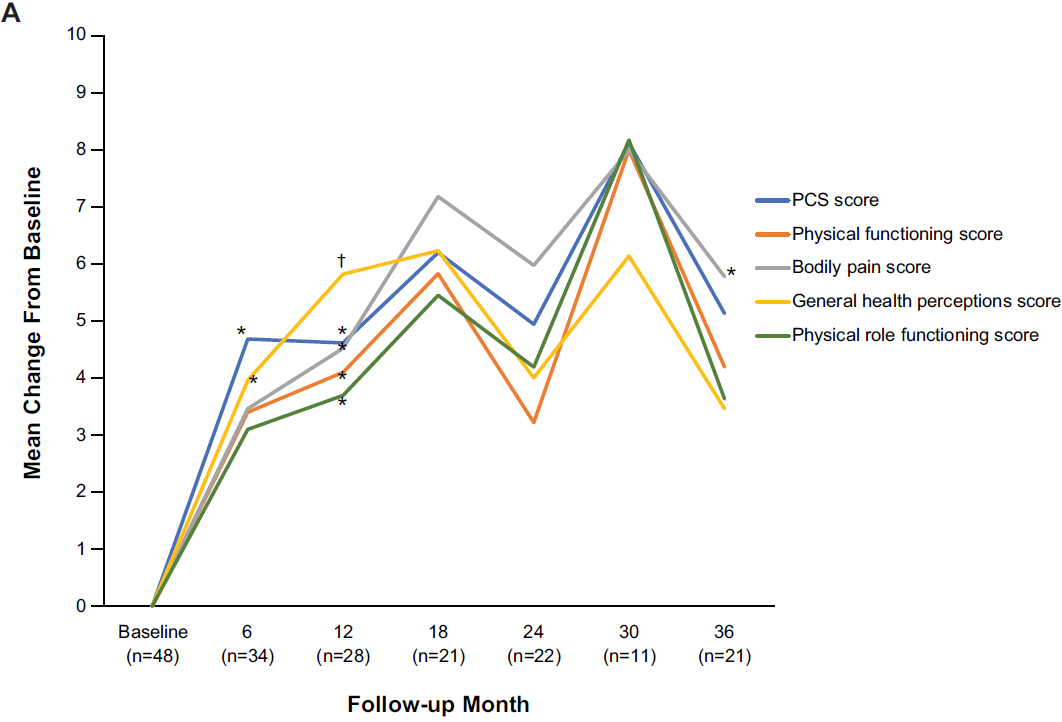


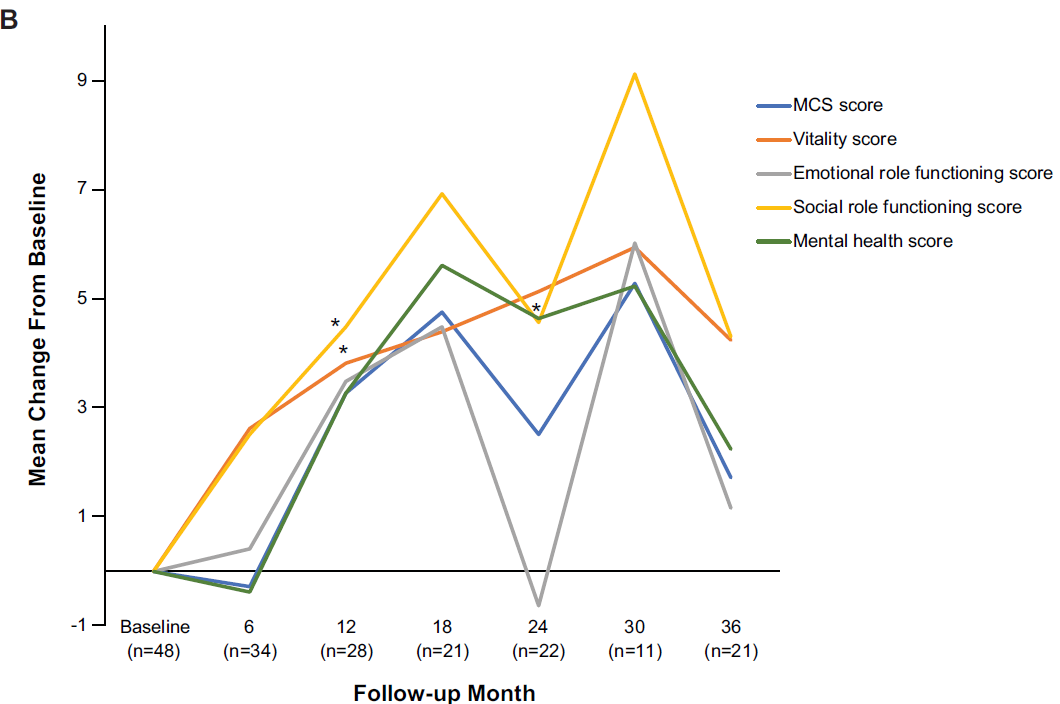


**P*≤0.05; ^†^*P*=0.002.

MCS, Mental Component Summary; PCS, Physical Component Summary; SF-36v2, 36-item Short Form Health Survey.

**Supplementary Table 1.** Characteristics of Individual Patients With Low ALP and Negative or No Genetic Test Result

| **Genetic status** | **Age at onset, years** | **ALP at baseline, U/L** | **PLP at baseline, nmol/L** | **Age at baseline, years** | **Signs and symptoms** | **Skeletal event(s)** | **Fracture location** |
| --- | --- | --- | --- | --- | --- | --- | --- |
| Negative | 6 | 26 | Not available | 55 | - Abnormal gait - Chronic bone pain - Chronic inflammation - Chronic muscle pain - Early loss of primary teeth - Fatigue - Generalized body pain - Hyperphosphatemia - Loose teeth - Loss of permanent teeth - Poor dentition - Pseudofractures - Recurrent and poorly healing fractures - Weakness | - Bone pain serious/severe enough to limit activities | - Femur (x3) - Vertebral column - Metatarsal |
| Not performed; refused by patient but positive family history | 10 | 26 | 454.5 (elevated) | 78 | - Loss of permanent teeth - Loose teeth - Poor dentition - Recurrent and poorly healing fractures | - Osteoarthritic features affecting the patellofemoral articulation - Loose body formation | - Pelvis - Humerus - Femur |
| Negative | 17 | 32^a^ | 8.9 | 64 | - Abdominal pain - Chronic bone pain - Chronic muscle pain - Depression symptoms - Esophageal reflux - Fatigue - Generalized body pain - Hypertension - Loose teeth - Recurrent and poorly healing fractures - Weakness | - Bone pain serious/severe enough to limit activities | - Multiple, bilateral, non-healing metatarsal for over 20 years - Sternum |
| Not performed; refused by patient | 1.5 | 17 | 92.7 | 31 | - Abdominal pain - Abnormal gait - Chronic bone pain - Dentures - Depression symptoms - Early loss of primary teeth - Fatigue - Generalized body pain - Hypertension - Loss of permanent teeth - Migraines - Nausea - Poor dentition - Severe insomnia - Weakness | - Scoliosis - Bone pain serious/severe enough to limit activities | - Ankle/malleolus - Hand |
| Not performed | 16 | 20 | 97.1 | 21 | - Abdominal pain - Abnormal gait - Chronic bone pain - Chronic inflammation - Chronic muscle pain - Chronic or repeated gastrointestinal symptoms - Complex aphthous - Depression symptoms - Early loss of primary teeth - Fatigue - Fibromyalgia - Generalized body pain - Irritable bowel syndrome - Nausea - Vomiting - Weakness | - No skeletal assessment performed | - None |
| Not performed | Pediatric onset | 11 | Not available | 70 | - Bone deformity - Chronic muscle pain - Craniosynostosis - Delayed walking - Early loss of primary teeth - Rickets - Seizures - Short stature - Waddling gait | - No skeletal assessment performed | - Femur (x2) |
| Not performed | 5 | 20 | Not available | 69 | - Bone deformity - Chronic muscle pain - Chronic or repeated gastrointestinal symptoms - Dentures - Depression symptoms - Early loss of primary teeth - Esophageal reflux - Fatigue - Hypertension - Loose teeth - Loss of permanent teeth - Nausea - Poor dentition - Recurrent and poorly healing fractures | - Scoliosis - Spinal spondylosis - Facet joint arthritis, lumbar spine | - Rib - Metatarsal (x2) - Femur (x2) - Tibia - Hand - Vertebral column |
| Result not available | 3.33 | 24 | 95 | 47 | - Abnormal gait - Acute intracranial pressure - Chronic bone pain - Chronic muscle pain - Cognitive delay/developmental delay - Early loss of primary teeth - Failure to thrive - Fatigue - Generalized body pain - Recurrent and poorly healing fractures - Weakness | - Unknown | - Femur |
| Not performed | 37 | 16 | N/A | 73 | - Abnormal gait - Chronic bone pain - Early loss of primary teeth - Hypertension - Loss of permanent teeth - Recurrent and poorly healing fractures | - Bowing of long bones in arms | - Wrist (x3) - Patella (x2) - Femur (x2) |

^a^Age- and sex-adjusted ALP values below normal for 8 years prior to genetic testing

ALP, alkaline phosphatase; PLP, pyridoxal 5ʹ-phosphate.
